# Allocation of forest biomass across broad precipitation gradients in China’s forests

**DOI:** 10.1038/s41598-018-28899-5

**Published:** 2018-07-12

**Authors:** Zhiyang Lie, Li Xue, Douglass F. Jacobs

**Affiliations:** 1 College of Forestry and Landscape Architecture, South China Agricultural University, Guangzhou, 510642 P. R. China; 2 Department of Forestry and Natural Resources, Hardwood Tree Improvement and Regeneration Center, Purdue University, West Lafayette, IN 47907-2061 USA

## Abstract

Forests act as major sinks for atmospheric CO_2_. An understanding of the relationship between forest biomass allocation and precipitation gradients is needed to estimate the impacts of changes in precipitation on carbon stores. Biomass patterns depend on tree size or age, making it unclear whether biomass allocation is limited by tree age at regional scales. Using a dataset of ten typical forest types spanning a large age scale, we evaluated forest biomass allocation–precipitation correlations with the aim of testing whether biomass allocation patterns vary systematically in response to altered precipitation. With increasing mean annual precipitation, a significant quadratic increase occurred in ≤30 yr and >60 yr groups in stem biomass, >60 yr group in branch biomass, and >60 yr groups in leaf biomass; and a significant cubic increase occurred in 30–60 yr and all age forest groups in stem biomass, ≤30 yr, 30–60 yr and all age forest groups in branch biomass, ≤30 yr and all age forest groups in leaf biomass, and in each group in root biomass, indicating that organ biomass is strongly limited by precipitation. Thus, forest biomass responds predictably to changes in mean annual precipitation. The results suggest that forest organ biomass–precipitation relationships hold across independent datasets that encompass a broad climatic range and forest age.

## Introduction

Over the past several decades, the earth has experienced profound climatic change^1,2^, which affects forest growth. Forest biomass has thus received increased attention^3^ due to its major sinks for atmospheric CO_2_. An understanding of the relationship between forest biomass and climate is needed to predict the impacts of climate change on carbon stores^4^. Plants can differentially allocate biomass to leaves, stems and roots depending on tree size or age in a stand^5^, which follow ontogenetic trajectories that interact with the prevailing climate^6^. Enquist and Niklas^7^ predicted scaling relationships among organ masses by a general allometric model based on metabolic theory, and Zhang *et al*.^8^ studied the organ biomass-density relationship in Chinese forests. However, little information is available regarding the influence of climate on organ biomass in forests across different stages of age. Plant biomass allocation patterns are important for global carbon cycling^9^. At the forest community level, plant biomass allocation strategies are linked to environmental change^10–13^. When different forest types are considered, results are controversial. Some researchers suggested that biomass distribution can be influenced by climate^6,12^, whereas Cairns *et al*.^14^ found no relationship between global root biomass and climate. Whether these allocation patterns vary systematically across climatic gradients remains unknown, which represents a particularly critical knowledge gap^15,16^.

Precipitation is a crucial environmental factor in determining ecosystem biomass^16^ because the direct and indirect influence of moisture availability is important to growth and productivity^17,18^. Altered precipitation associated with climatic change is significantly altering the forest biomass of terrestrial ecosystems^2^. Consequently, studying forest responses to large-scale spatial variation in precipitation can strengthen our understanding of the mechanisms of ecosystem adaptation and response to climate change^19^. A growing number of studies have proposed that annual precipitation governs forest biomass^20^, but many forest biomass studies are based on empirical correlations restricted to a single forest type^21^. Although responses to precipitation were confirmed in some biomass patterns in these studies, whether the results of these studies can be extrapolated to a larger geographic scale remains unclear^4^. Moreover, many studies of large-scale precipitation variability have focused solely on shoot and root biomass, primarily root:shoot ratios of individual species or plant communities under specific environmental conditions^22^. Few studies have examined the relationship of biomass among organs, such as stem, branch, leaf and root, with precipitation. Consequently, it is often unclear how the relative balance among organ biomass varies across a precipitation gradient. Such information is necessary for fine-tuning or constraining carbon stock estimates by vegetation type and precipitation zone^23^ and for improving our understanding of how precipitation affects forest biomass^4^.

China has forest types across broad geographic regions and environmental gradients^24^, which vary across a wide range of climatic regimes. These types include temperate and subtropical forests across North, Central and South China. Because of the wide distribution of these forests, there is a steep latitudinal gradient of precipitation for each type. Despite the importance of these forests for carbon cycling in China, influences of precipitation on biomass allocation among organs in these forests remain unclear.

Biomass allocation in young forests may be different from older forests. Thus, one approach to understand precipitation effects on biomass allocation in different growth stage forests is to compare biomass allocation patterns in forests of varying age along natural precipitation gradients. Data from across China provide an important opportunity to examine biomass allocation in relation to tree age and precipitation, which may contribute valuable insight toward understanding precipitation effects on carbon sequestration in forests. The objective of the present study is to test whether biomass allocation patterns of different age groups vary systematically across a mean precipitation gradient. Although many other environmental and climatic factors affect biomass allocation, we focused our analysis on mean annual precipitation (MAP) because of the availability of comprehensive data on this variable.

## Results

**Figure 1:**
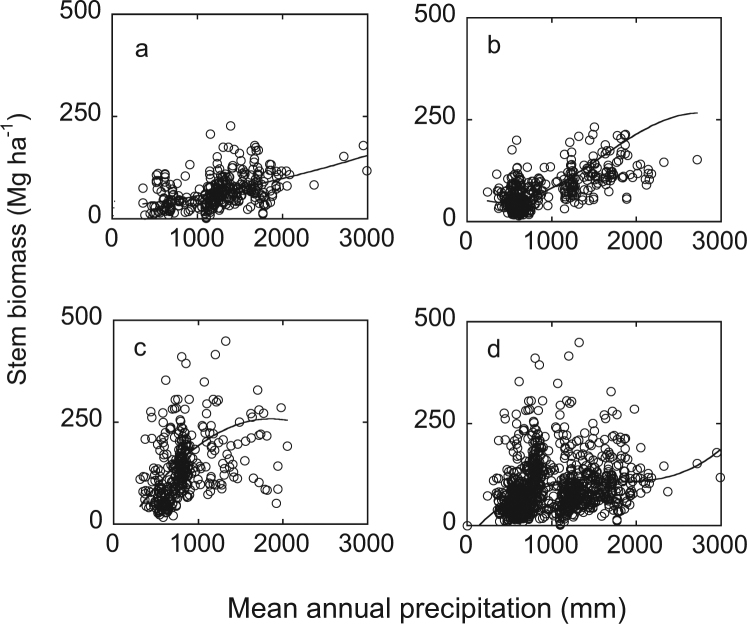
Relationship between mean annual precipitation and stem biomass along a precipitation gradient in China. (**a**) ≤30 yr; (**b**) 31–60 yr group; (**c**) >60 yr group; (**d**) all age forests.

Organ biomass had a quadratic or cubic response pattern to precipitation change. With MAP, stem biomass had a significant quadratic increase in ≤30 yr and >60 yr groups, and a significant cubic increase in 30–60 yr and all age groups (*P* < 10^−6^) (Table 1, Fig. 1). Adjusted R^2^ for the two equations was ≥0.18.

**Table 1:** The best models for organ biomass of different age groups (quadratic: y = C + b1x + b2x2 and cubic: y = C + b1x + b2x2 + b3x3) and associated equations for data of forest-level organ biomass (stem biomass *w*_s_, Branch biomass *w*_B_, leaf biomass *w*_L_ and root biomass *w*_R_) (Mg ha^−1^) and mean annual precipitation MAP (mm).

Organ	Forest age group	Equation	Model Summary	*P*	C	Parameter Estimates	b2	b3
			Adjusted R^2^			b1		
Stem	≤30 yr	Quadratic	0.18	<10^−6^	28.6	0.021	7.160E-06	—
	31–60 yr	Cubic	0.50	<10^−6^	71.5	−0.118	0.000165	−0.000000044
	>60 yr	Quadratic	0.18	<10^−6^	−50.3	0.333	−0.000113	—
	all age forests	Cubic	0.18	<10^−6^	−32.7	0.257	−0.000157	3.18E-08
Branches	≤30 yr	Cubic	0.12	<10^−6^	17.5	−0.028	2.95E-05	−6.85E-09
	31–60 yr	Cubic	0.50	<10^−6^	23.6	−0.0520	6.71E-05	−1.73E-08
	>60 yr	Quadratic	0.41	<10^−6^	20.4	−0.026	3.31E-05	—
	all age forests	Cubic	0.19	<10^−6^	24.7	−0.043	4.84E-05	−1.14E-08
Leaves	≤30 yr	Cubic	0.23	<10^−6^	−1.8	0.015	−9.75E-06	2.60E-09
	31–60 yr	Quadratic	0.33	<10^−6^	4.7	−0.0007	2.62E-06	−2.68E-10
	>60 yr	Quadratic	0.17	<10^−6^	1.8	0.010	−1.68E-06	—
	all age forests	Cubic	0.18	<10^−6^	−0.4	0.016	−1.10E-05	2.92E-09
Roots	≤30 yr	Cubic	0.20	<10^−6^	32.9	−0.046	3.30E-05	−5.11E-09
	31–60 yr	Cubic	0.26	<10^−6^	29.0	−0.036	4.15E-05	−9.76E-09
	>60 yr	Cubic	0.18	<10^−6^	19.6	−0.015	8.09E-05	−3.39E-08
	all age forests	Cubic	0.14	<10^−6^	2.3	0.057	−3.88E-05	9.09E-09

Data, grouped according to forest age, taken from Luo (1996) and others.

**Figure 2:**
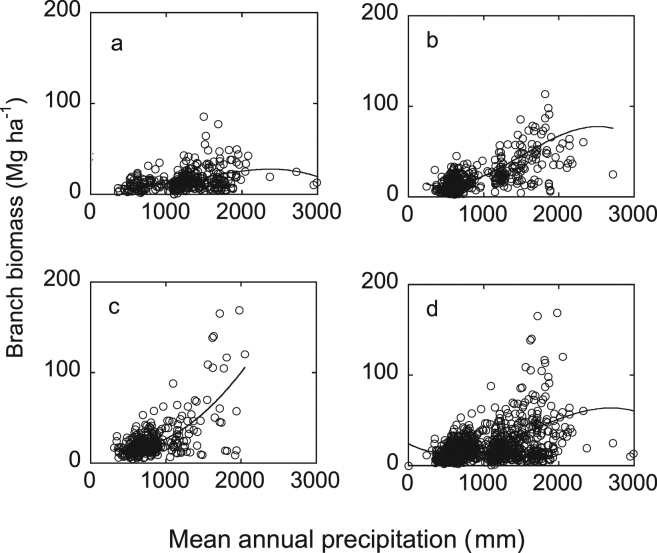
Relationship between mean annual precipitation and branch biomass along a precipitation gradient in China. (**a**) ≤30 yr; (**b**) 31–60 yr group; (**c**) >60 yr group; (**d**) all age forests.

Branch biomass had a significantly positive cubic correlation with MAP in ≤30 yr, 30–60 yr and all age group groups, and a quadratic correlation in >60 yr group (*P* < 10^−6^) (Fig. 2). Adjusted R^2^ for all equations was ≥0.12.

**Figure 3:**
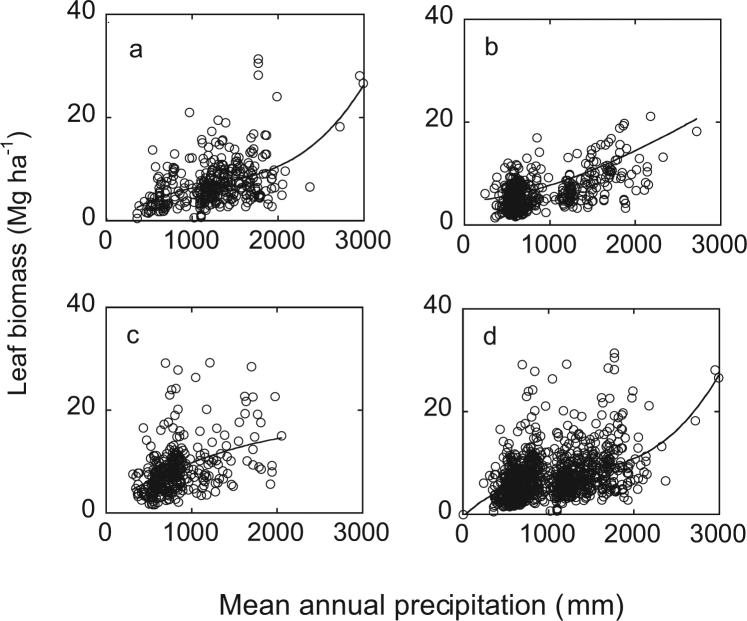
Relationship between mean annual precipitation and leaf biomass along a precipitation gradient in China. (**a**) ≤30 yr; (**b**) 31–60 yr group; (**c**) >60 yr group; (**d**) all age forests.

Leaf biomass showed a significant cubic correlation with MAP in ≤30 yr and all age groups, and a quadratic correlation with MAP in 30–60 yr and >60 yr groups (*P* < 10^−6^) (Fig. 3). Adjusted R^2^ for all equations was ≥0.17.

**Figure 4:**
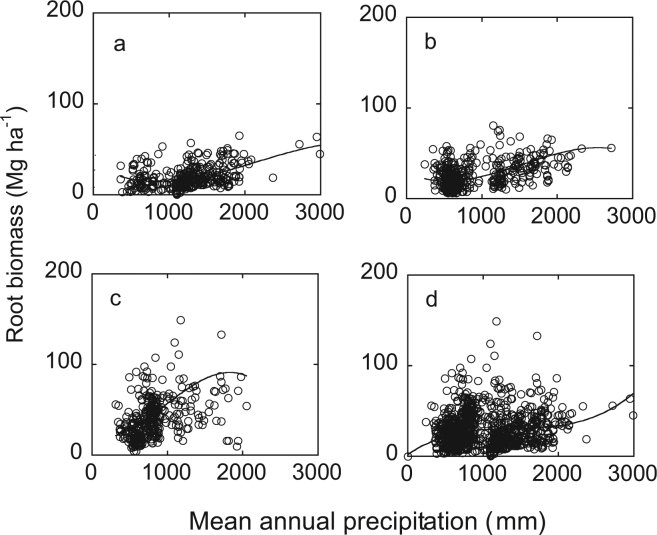
Relationship between mean annual precipitation and root biomass along a precipitation gradient in China. (**a**) ≤30 yr; (**b**) 31–60 yr group; (**c**) >60 yr group; (**d**) all age forests.

Root biomass significantly cubic increased in ≤30 yr, 30–60 yr, >60 yr and all age groups (*P* < 10^−6^) (Fig. 4). Adjusted R^2^ for all equations was ≥0.14.

Adjusted R^2^ were characterized by this sequence: stem >branches >leaves >roots. The lower R^2^ values for leaves and roots may reflect the smaller sample sizes for these organs. When predicting tree biomass, stem biomass is more stable than that of more short-lived leaves.

## Discussion

Forest organ biomass varies across a large scale as a result of precipitation gradients^25^. Certain past studies have analyzed patterns of forest biomass in response to large-scale precipitation changes in China^26–28^. However, most of these have focused on temperate forests, with few analyzing forest biomass allocation in subtropical regions. In this study, we showed that stem, branch, leaf and root biomasses increased significantly with increasing MAP in each age group. Saatchi *et al*.^29^ reported that total aboveground biomass increases with precipitation in moist and wet tropical forests of the Amazon Basin. McCarthy and Enquist^30^ found that stem mass increased with increased precipitation. These reports, together with our results, suggest that precipitation gradient is the critical variable driving forest biomass within these forest types. Greater annual precipitation is presumably favorable for vegetative shoot growth over the growing season^31^. Reduced precipitation lowers nutrient availability due to water limitation of soil microbial processes, attenuates ecosystem photosynthesis, and ultimately results in an overall decrease of biomass and productivity^32,33^.

Increasing root biomass favors uptake of nutrients and water^34,35^. Despite greater attention over recent decades, knowledge of root biomass and its spatial distribution is much more limited than that of shoot biomass^36^. Further, root dynamics remain largely uncertain^35^ because there have been few studies on responses of root biomass to spatial variations in precipitation^37^. Our results show that root biomass was significantly related to MAP in each age group (cubic model), which indicates that increased precipitation promotes accumulation of root biomass of Chinese forests.

Plant ontogeny may also have a strong effect on biomass allocation patterns^38^. With tree growth, wood (dead cells) continuously accumulates in stems and roots, whereas branch and leaf biomass decrease because crown growth is impeded and shade-intolerant leaves shed at the canopy base. Thus, emphasis in biomass allocation transitions over time from leaves and branches to stem and root^39,40^. However, our analyses indicate that precipitation is consistently correlated with biomass allocation in forests of varying age stages. When data from forests of all ages were considered together, forest organ biomass was also correlated with MAP, which suggests that the decisive role of precipitation is consistent across forest age. As a result, it is possible to make general predictions concerning changes in biomass allocation in response to precipitation change, which may prove important for understanding forest biomass allocation patterns in the context of changing precipitation regimes.

Our data indicate that precipitation affects organ biomass allocation patterns, which indicates that the role of precipitation in determining biomass allocation is dependent on forest age, which is in line with the result of Zhang *et al*.^12,41^ who found that forest biomass varies with climatic variables, such as precipitation and temperature. Moreover, Reich *et al*.^16^ and Lie and Xue^42^ reported that organ biomass are also associated with temperature gradients, which implies that allocation patterns of organ biomass vary systematically as a result of climatic gradients, and will be useful for assessing climate change impacts on forest carbon stocks and cycles. By focusing on forest organ biomass, we have provided evidence consistent with a causal link between precipitation and forest biomass allocation. Low precipitation appears to limit all organ biomass associated with its effect on embolism and hydraulic conductance of individual trees^4^. In view of climate change, more frequent changes in precipitation may occur due to increasing frequency and severity of drought, as well as greater occurrence of floods and extreme weather. Additional studies that evaluate the influence of precipitation on forest biomass allocation at a global scale will be critical for understanding whether these regional-scale patterns may be applied at broad geographic scales and how changes in precipitation will alter future terrestrial carbon stores.

In conclusion, this study examined biomass allocation patterns of forests in different age stages across China along precipitation gradients. With increasing MAP, organ biomass showed a significant quadratic increase in ≤30 yr and >60 yr groups for stem and in >60 yr group for branches and leaves; and a significant cubic increase in 30–60 yr and all age groups for stem; in ≤30 yr, >60 yr and all age groups for branches and leaves; and in ≤30 yr, 30–60 yr, >60 yr and all age groups for roots. Forest organ biomass–precipitation relationships hold across independent datasets that encompass a broad climatic range and forest age. These results could be used to predict precipitation influences on standing biomass. Such predictions will be essential for understanding feedbacks between climate change and forest community carbon storage.

## Materials and Methods

### Dataset

A 1193-large organ biomass and climatic dataset was compiled from the literature, in which 916 data points were extracted from the database of Luo^43^. These originated from the inventories of the Forestry Ministry of China, and others collected from 21 sources^44–63^. These data include alpine temperate *Larix* forest, alpine *Picea-Abies* forest, temperate typical deciduous broadleaved forest, temperate *Pinus tabulaeformis* forest, temperate mixed coniferous-broadleaved forest, montane *Populus-Betula* deciduous forest, subtropical evergreen broadleaved forest, subtropical montane *Cupressus* and *Sabina* forest, subtropical *Pinus massoniana* forest and subtropical *Cunninghamia lanceolata* forest. These are typical forest types from north to south in China and represent all the major forest types in China^43^. These forest types are widely distributed land-cover types covering an area of 114.3 million ha, occupying about 80% of Chinese forest area^42,64^.

Forest biomass of trees was measured by destructive harvesting in experimental plots. The size of these plots varied with stand condition and forest type but in general, the area of sample plots ranged from 400–1000 m^2^ for each forest.

For each data point, we documented the following information, if available: (1) stem, branch, leaf, root and total biomass; (2) diameter-at-breast height (DBH) and tree height. The sampled forests varied widely in size (with total forest biomass ranging from 3.3 to 657.3 Mg/ha) and in forest age (from 3 to 261 years). The sampled forests varied widely geographically (81°00′–134°00′N, 18°70′–52°60′E), in mean annual temperature (from −6.6 to 24.2 °C) and MAP (from 241.0 to 2989.1 mm).

Because of changes in organ biomass allocation with forest age, the data of the ten forests was compiled according to forest age, which was obtained from data^43^ and classified as ≤30 yr (347 data points), 31–60 yr (514 data points) and >60 yr (362 data points) based on forest age.

### Model development and selection

Biomass modelling was performed using SPSS (SPSS 19.0 (SPSS Inc., Chicago, IL, USA, 2004) and eviews (eviews 8.0, Quantitative Micro Softwar, USA). Generally, biomass allocation displays a linear relationship with precipitation^12,13,65,66^, though sometime it shows a curvilinear relationship with MAP^12,13^. In the present study, linear and curvilinear (quadratic or cubic) equations were used to fit data. Akaike Information Criteria (AIC) was proposed to overcome the inherent weaknesses of traditional variable selection methods, such as stepwise selection, forward and backward elimination^67^; therefore, it is a useful tool for model selection where the model with the lowest AIC indicates the best model^68^. This criterion is based on Akaike information theory and has been widely used in variable and model selection^68^. To compare models, the ΔAIC was calculated as the difference in AIC values between each model and the model with the lowest AIC^69^ with smaller values indicating better fit (the best fitting model would have ΔAIC = 0 and all other models ΔAIC >0)^70^, and then higher adjusted *R*^2^ values were considered. According to the ΔAIC results, quadratic or cubic equations fit better than linear equations against organ biomass data (Supplementary Table 1).

## Electronic supplementary material

Dataset 1
