# Supplementary material for: Allocation of forest biomass across broad precipitation gradients in China’s forests
Source: Sci Rep. 2018 Jul 12;8:10536. doi: 10.1038/s41598-018-28899-5 (PMC6043570; doi:10.1038/s41598-018-28899-5)
Supplement: Supplementary file 1 — Dataset 1 [file 41598_2018_28899_MOESM1_ESM.docx]

Supplementary Materials:

**Title: Allocation of forest biomass across broad precipitation gradients in China’s forests**

**AUTHORS**: Zhiyang Lie^1^, Li Xue^1^, Douglass F. Jacobs^2^

**AFFILIATIONS**: ^1^College of Forestry and Landscape Architecture, South China Agricultural University, Guangzhou, 510642, P. R. China; ^2^Department of Forestry and Natural Resources, Hardwood Tree Improvement and Regeneration Center, Purdue University, West Lafayette, IN 47907-2061, USA

**Table 1. ΔAIC of various models used in analyses. Models were evaluated for the difference in AIC between each model and the lowest value of AIC obtained (ΔAIC).  The models with the smallest ΔAIC** **(ΔAIC= 0) were deemed the best models.**

| Organ |  | ΔAIC | | |
| --- | --- | --- | --- | --- |
|  | Forest age group | Linear | Quadratic | Cubic |
| Stem | ≤ 30 yr | 1.481 | 0 | 0.002 |
|  | 31-60 yr | 0.034 | 0.034 | 0 |
|  | > 60 yr | 1.501 | 0 | 0.003 |
|  | all age forests | 0.014 | 0.008 | 0 |
| Branches | ≤ 30 yr | 0.906 | 0.01 | 0 |
|  | 31-60 yr | 0.032 | 0.035 | 0 |
|  | > 60 yr | 0.674 | 0 | 0.003 |
|  | all age forests | 0.014 | 0.015 | 0 |
| Leaves | ≤ 30 yr | 0.009 | 0.016 | 0 |
|  | 31-60 yr | 0.01 | 0 | 0.004 |
|  | > 60 yr | 0.957 | 0 | 0.004 |
|  | all age forests | 0.022 | 0.018 | 0 |
| Roots | ≤ 30 yr | 1.083 | 0.006 | 0 |
|  | 31-60 yr | 0.007 | 0.008 | 0 |
|  | > 60 yr | 1.445 | 0.005 | 0 |
|  | all age forests | 0.004 | 0.006 | 0 |
